# Supplementary material for: Predicting the antigenic evolution of SARS-COV-2 with deep learning
Source: Nat Commun. 2023 Jun 13;14:3478. doi: 10.1038/s41467-023-39199-6 (PMC10261845; doi:10.1038/s41467-023-39199-6)
Supplement: Supplementary file 1 — Supplementary Information File [file 41467_2023_39199_MOESM1_ESM.pdf]

Supplementary Information for

Predicting the antigenic evolution of SARS-COV-2 with deep learning

Wenkai Han[1,2,#], Ningning Chen[1,2,#], Xinzhou Xu[3,4,#], Adil Sahil[1,2], Juexiao Zhou[1,2], Zhongxiao Li[1,2], Huawen Zhong[2], Elva Gao[5], Ruochi Zhang[6], Yu Wang[6], Shiwei Sun[7,8,*], Peter Pak-Hang Cheung[3,4,*], Xin Gao[1,2,*]

[1]Computer Science Program, Computer, Electrical and Mathematical Sciences and Engineering Division, King Abdullah University of Science and Technology (KAUST), Thuwal 23955-6900, Kingdom of Saudi Arabia
[2]Computational Bioscience Research Center, King Abdullah University of Science and Technology, Thuwal 23955-6900, Kingdom of Saudi Arabia
[3]Department of Chemical Pathology, Faculty of Medicine, Chinese University of Hong Kong, Hong Kong, China
[4]Li Ka Shing Institute of Health Sciences, Chinese University of Hong Kong, Hong Kong, China
[5]The KAUST School, King Abdullah University of Science and Technology (KAUST), Thuwal 23955-6900, Kingdom of Saudi Arabia
[6]Syneron Technology, Guangzhou, 510000, China
[7]Key Lab of Intelligent Information Processing, Institute of Computing Technology, Chinese Academy of Sciences, Beijing, 100190, China
[8]University of Chinese Academy of Sciences, Beijing 100049, China
[#]The first three authors contributed equally to this paper.
*All correspondence should be addressed to X.G. (xin.gao@kaust.edu.sa), P.C. (ppcheung@cuhk.edu.hk), and S.S. (dwsun@ict.ac.cn).

Contents

Supplementary Note 1 | In silico estimation of variant sequence risk.
Supplementary Note 2 | The reason we convert the continuous values into binary labels.
Supplementary Fig. 1 | The distribution of the DMS scores of eight antibodies.
Supplementary Fig. 2 | Five-fold cross validation comparison.
Supplementary Fig. 3 | Benchmark feature encoding methods.
Supplementary Fig. 4 | Ablation studies.
Supplementary Fig. 5 | MLAEP predictions against a pseudovirus neutralization test dataset.
Supplementary Fig. 6 | Pseudo time and ESM-1b model inference.
Supplementary Fig. 7 | Two-dimensional contour plot comparison.
Supplementary Fig. 8 | Correlation between the model scores and sampling date.
Supplementary Fig. 9 | Principal component analyses of the sequence's representations from our model.
Supplementary Fig. 10 | 3D structure of RBD - ACE2 complex.
Supplementary Fig. 11 | KL logo plot for the entire RBD region.
Supplementary Fig. 12 | Eight RBD mutants bearing different mutations on the surface were selected for binding assay against monoclonal antibodies.
Supplementary Fig. 13 | Overview of the synthetic sequences and GISAID sequences.
Supplementary Fig. 14 | 3D views of docking simulation results.
Supplementary Fig. 15 | Evaluate the risks of the synthetic sequences.
Supplementary Fig. 16 | Box plot for the existing GISAID sequences and the synthetic sequences.
Supplementary Fig. 17 | The difference among the initial sequences and the synthetic sequences.
Supplementary Table 1 | Baseline model performance comparison.
Supplementary Table 2 | Validate model performance on extra datasets.
Supplementary Table 3 | Model performance evaluation on antibody neutralization dataset.
Supplementary Table 4 | Model predicted mutations was also found in immunocompromised patients.
Supplementary Table 5 | In vitro validation experiments selection criteria.

Supplementary Note 1 | In silico estimation of variant sequence risk.

Having generated the synthetic sequences and their mutational sites, we next evaluated the risk of the variant sequences with Evo-velocity analysis and viral language model risk inference, followed by structure modeling and antibody-antigen docking.

We first visualized the synthetic sequences together with the existing GISAID variant RBD sequences with the Evo-velocity figure (Supplementary Fig. 13, 15 a,b). As the initial sequences for the generation mainly come from the Omicron lineage, our synthetic sequences and Omicron variants locate in a different cluster compared to the wild type and other VOCs. The streamlines suggest that the B.1.1.529 and the BA.2 may develop into different sublineages. Considering that the inferred pseudotime and trajectory have a high correlation on the existing GISAID variant sequences, the newly generated synthetic sequences, especially the ones located at the directional flow after B.1.1.529 and BA.2, need to be monitored as they have a high chance of becoming dominate variants in the coming wave.

The language models trained with the spike protein sequences with a self-supervised masked language modeling objective have been used to assess the risk of a viral variant. The risk is composed of two proxies, namely grammaticality as a fitness measurement and semantic for antigenic variation. We validated the synthetic and GISAID sequences in terms of the two dimensions. Compared with the existing covid variant sequences, the variants searched by our model have a larger semantic score, suggesting a high immune escape potential (Supplementary Fig.15c, 16, 17, Supp Table 7). While for the grammaticality dimension, the synthetic variants have lower variance and are generally high. Taken the two dimensions together, the synthetic variants are more likely to induce immune escape.

We further performed *in silico* antibody-antigen docking experiments to access the immune escape, which includes four antibodies that correspond to the four antibody epitope classes. We picked 20 synthetic variants RBD sequences with the highest average model prediction score and used the Swiss-model protein structure homology modeling server to get the variants' structure. To calculate the binding score, we used the difference of estimated binding energy between the bound complex and the sum of unbounded isolates. We performed 1000 independent runs with the SnugDock for each antibody-antigen pair (Supplementary Fig. 14, 15d) and used the mean value as a proxy for binding score (Supplementary Fig. 15e). We also performed similar experiments for Wuhan-wildtype and the Omicron variants for comparison. The synthetic variants perform generally higher binding scores compared with that of the wild type, while compared with the Omicron variant, the synthetic sequences perform as well as (or even higher immune escape potential than) Omicron. The simulated docking experiments also suggest that the generated variants have a high probability of becoming immune escape.

Supplementary Note 2 | The reason we convert the continuous values into binary labels.

As discussed in the paper, the entire machine learning module serves as a scoring function for the downstream searching part. There are several reasons that led us to take the classification option:

1. Interpretability. Binary labels are easier to interpret and can provide more clear-cut results.
2. Equalization. The range of the DMS values across experiments varies. For example, the label distribution of COV2-2832 ranges from 0 to 12, while the label distribution of COV2-2499 ranges from 0 to 9. We used a multi-task model to learn all tasks simultaneously and treat each task equally. In this case, using the raw value may affect our model performance because the batch effect will become a serious issue. Converting continuous values into binary labels would make each class comparable when performing the multitask learning. It could prevent batch effects and other variations introduced by different experiments.
3. Objective. We understood that a classification model may not provide a quantitative measure of the effect of mutations. However, our goal was to develop a model that could predict the directionality of the effect (i.e., whether a mutation increases or decreases binding affinity) rather than the magnitude of the effect.
4. Simplicity. Binary labels are easier to work with in our problem settings. Our label distribution in the extended Data Fig. 1 exhibits imbalanced distributions, where certain target values have significantly fewer observations. Compared with imbalanced classification problem, imbalanced regression learning is a challenging problem with limited efficient solutions. While existing solutions for learning from imbalanced data focus on targets with categorical indices, less attention was drawn on the deep imbalanced regression problem with continuous targets. To date, only a few deep learning algorithms have been proposed for this problem, and their effectiveness on biological data, particularly deep mutational scanning data, has not been thoroughly tested. We tested various models for regression, including our own, ESM model, CNN, RNN, LSTM, and traditional models like random forest and SVM. However, all models achieved poor performance on the problem, with the best spearman correlation coefficient of only about 0.2 for the antibodies and >0.8 for ACE2 binding. It suggests that predicting the ACE2 binding is relatively easy (it could be done in zero-shot settings with a protein language model), but the antibody binding affinity prediction is a much more challenging task. Our results are consistent with those of a newly published NeurIPS 2022 AI4Science workshop paper[1], which suggests the need for more efficient algorithms for deep mutational scanning regression. While our goal is not primarily focused on proposing such algorithms, we chose to transform the labels into categorical ones and use imbalanced classification algorithms to optimize our model for better performance in predicting the antigenic evolution. In this case, our goal was to train a model that could predict the directionality of the effect (i.e., whether a mutation increases or decreases binding affinity) rather than the magnitude of the effect.
5. Classification aligns with the genetic algorithm objective. We used the genetic algorithm to find possible mutations that changed the parents' label after several generations, in order to identify potential antigenic evolution. In this case, using a classification model

aligns well with the classification objective of the genetic algorithm. On the other hand, using a regression model could be more complex, as it would require defining a specific threshold for the genetic algorithm to stop. Therefore, it is more practical to use a classification model in combination with the genetic algorithm in this scenario.

6. Acceptability. Binarization, or the conversion of continuous values into binary labels, is a common practice in deep mutational scanning studies. Previous research on engineering monoclonal HER2 antibodies and analyzing RBD mutational profiles have also used binarization as a preprocessing step, indicating its acceptability in these types of datasets.

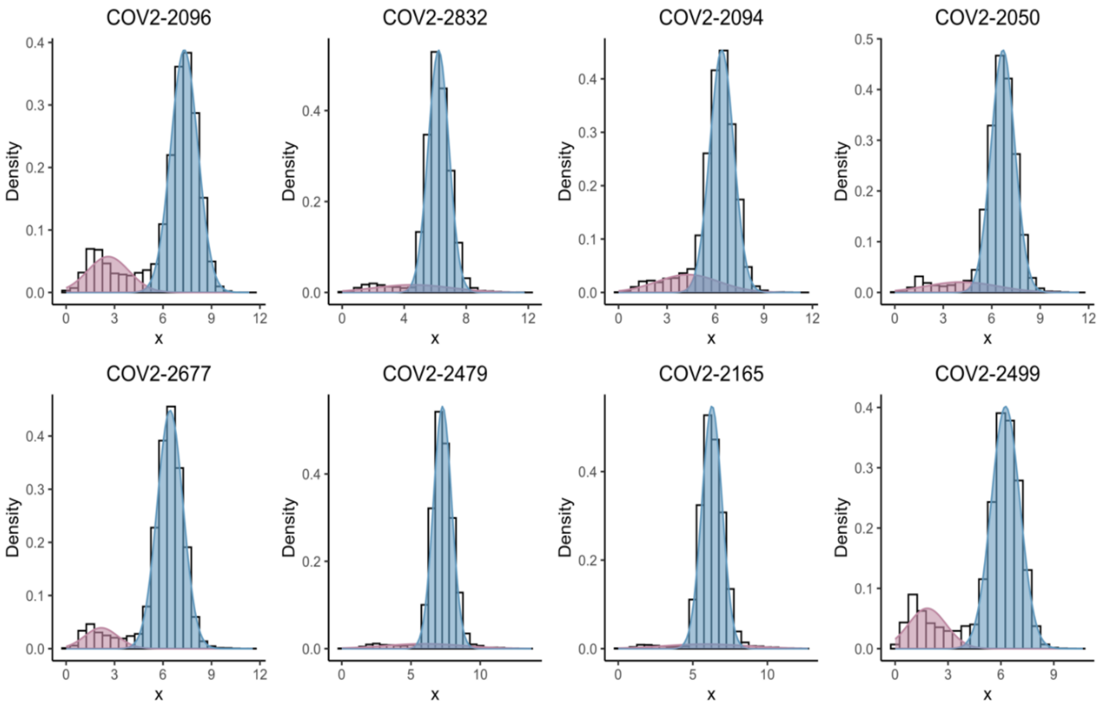

Supplementary Fig. 1 | The distribution of the DMS scores of eight antibodies. We log-transformed the deep mutational scanning scores, and got clearly two clusters for all antibodies. We then used the Gaussian mixture model to split the score into two clusters. Red cluster is defined as non-escape, while the blue clusters is defined as escape.

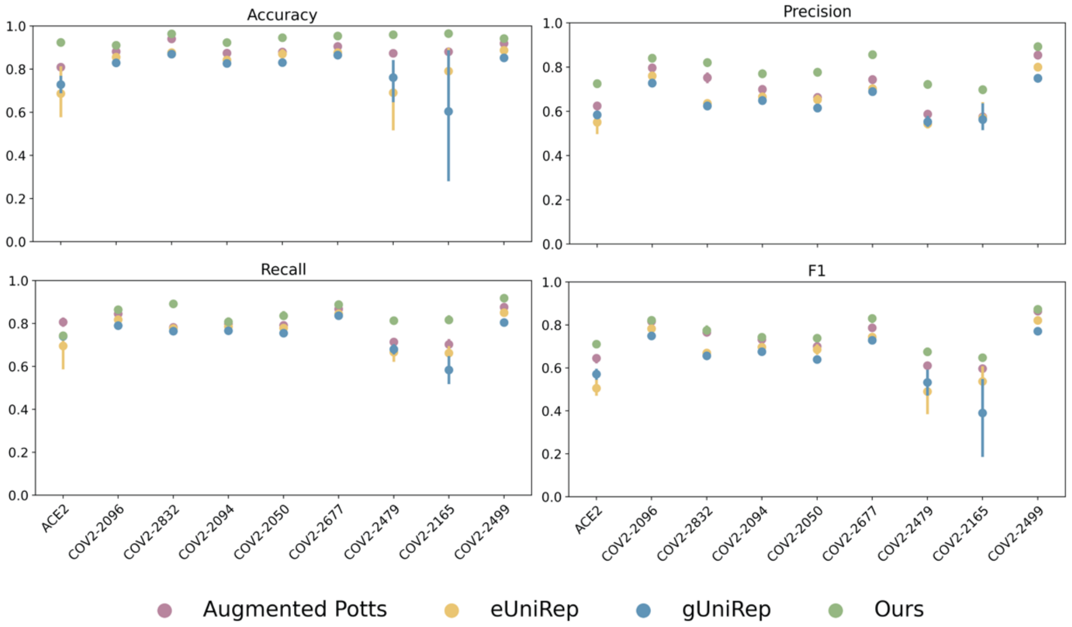

Supplementary Fig. 2 | Five-fold cross validation comparison. Performance of augmented Potts model, eUniRep model, gUniRep model, and our model for predicting ACE2 binding, and antibody escape is shown in terms of accuracy, macro precision, macro recall, and macro F1 score. The error bars represents the mean±standard deviation over five folds (n=5).

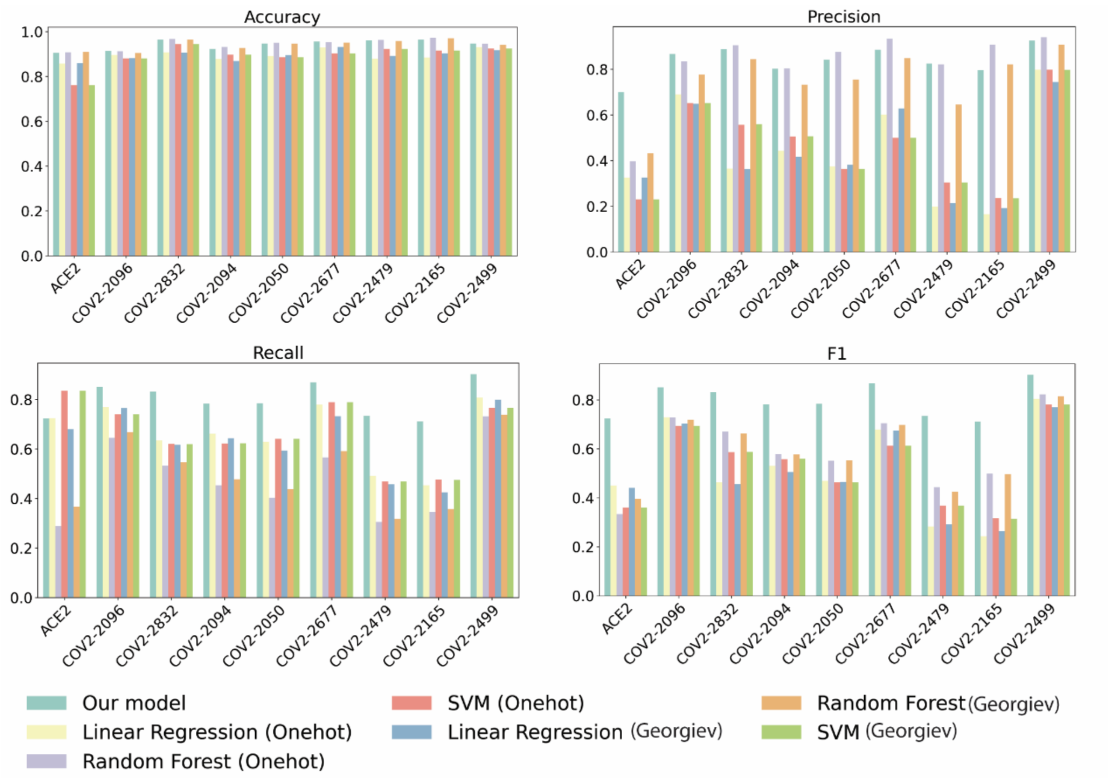

Supplementary Fig. 3 | Benchmark feature encoding methods. Performance of Onehot encoding and Georgiev encoding with traditional machine learning models, including SVM, Random Forest and Linear Regression. We also benchmarked our model for comparison. We did not find much improvement with the Georgiev encoding method against Onehot encoding.

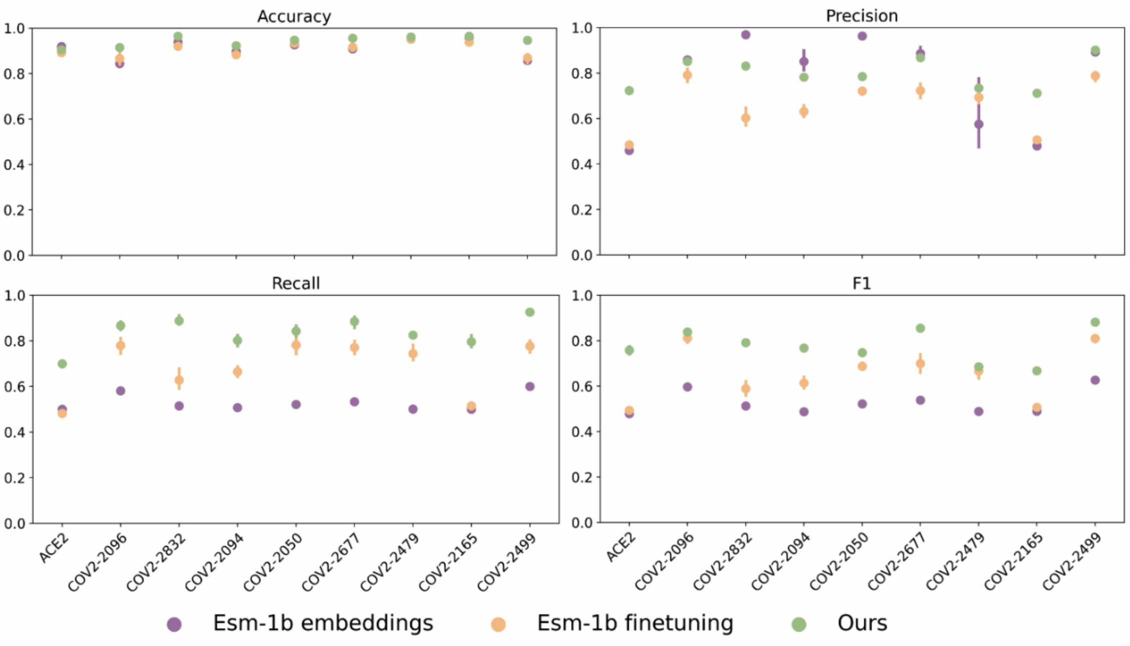

Supplementary Fig. 4 | Ablation studies. Justification of using the fine-tuning, the structure representations in the multi-task learning framework, in terms of Accuracy, macro-Precision, macro-Recall and macro-F1 score. ESM-1b shows the results of the fine-tuning steps' ablation.

ESM-1b finetuning shows the results of replacing the Structured Transformer's ablation. The error bars represents the mean±standard deviation over five folds (n=5).

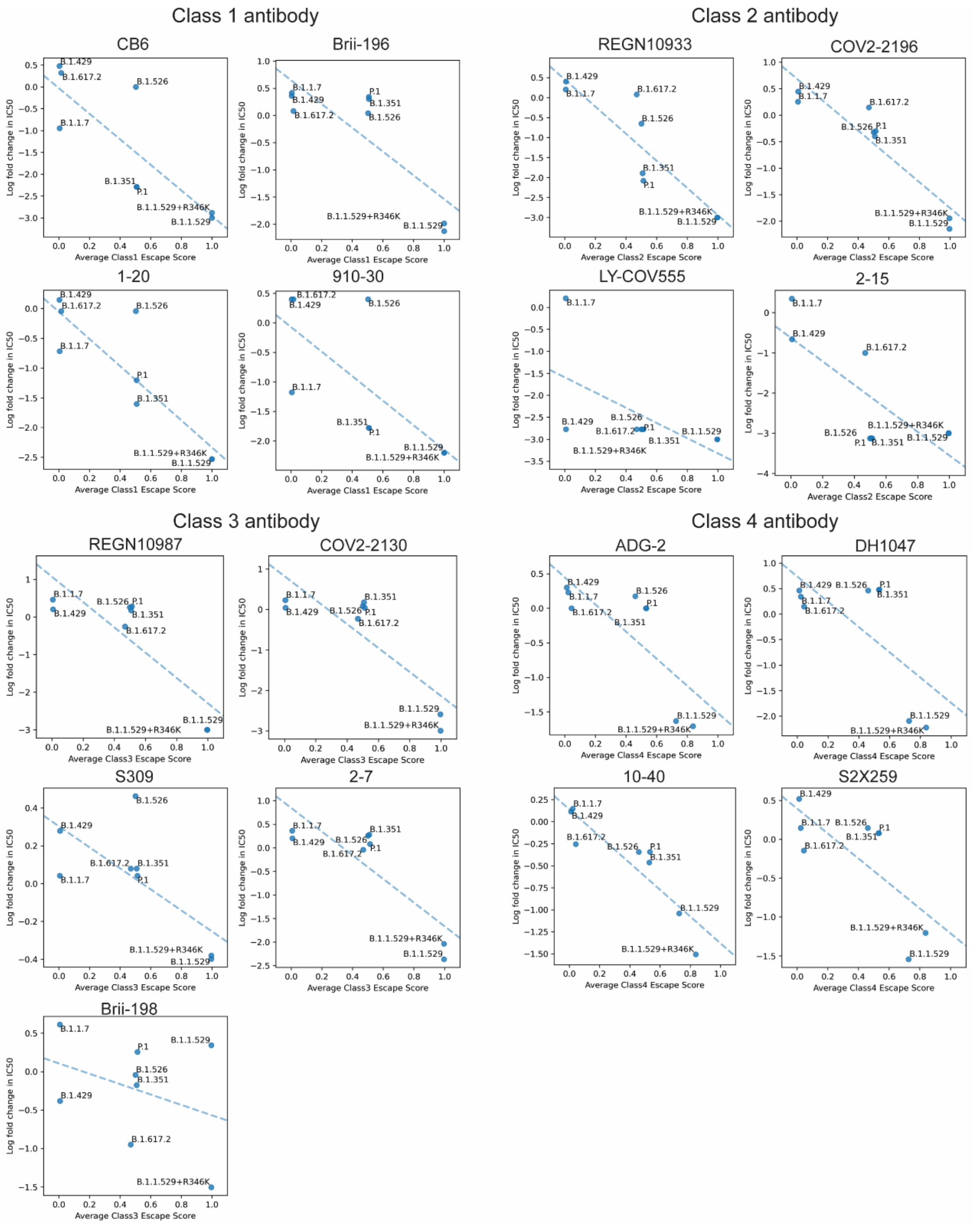

Supplementary Fig. 5 | MLAEP predictions against a pseudovirus neutralization test dataset. The dataset measured the cross-neutralizing effect of 17 RBD monoclonal antibodies against pseudoviruses expressing the Spike protein of selected variants of concern (VOCs). Validation of the predicted immune escape potential using the class specific monoclonal antibody-based pVNT assay data. The x-axis indicates the model predicted variant escape potential, while the y-axis is the log fold change pVNT50 reduction of the VOCs compared with the wildtype.

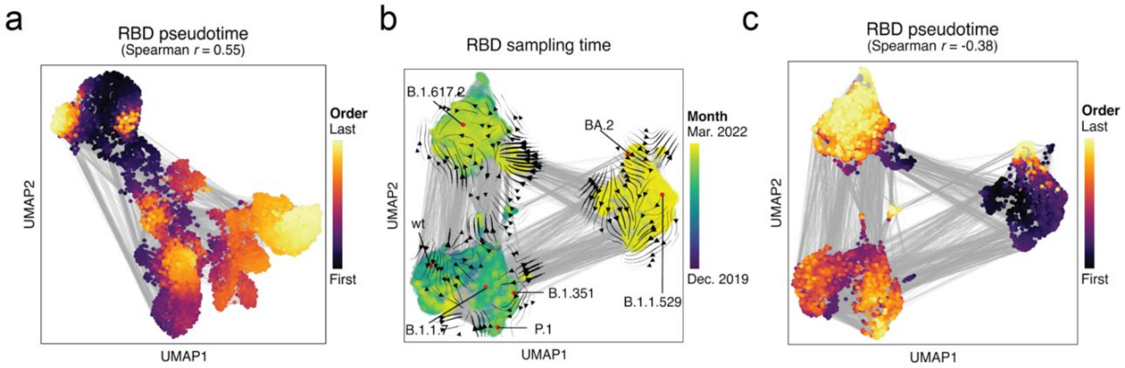

Supplementary Fig. 6 | Pseudo time and ESM-1b model inference. a, The landscape of RBD sequences, represented as a KNN network and visualized use the UMAP, colored with the inferred pseudo time using our model embeddings and scores. Gray lines indicate network edges. b and c, The landscape of the RBD sequences from GISAID, represented as a KNN similarity network and visualized with the UMAP. The sequences embeddings and directions among time are from the ESM-1b model. b, colored with the real-world sampling time. Streamlines show the visual correlation between the ESM-1b inferred velocity and sampling time. c, colored by the inferred pseudo time.

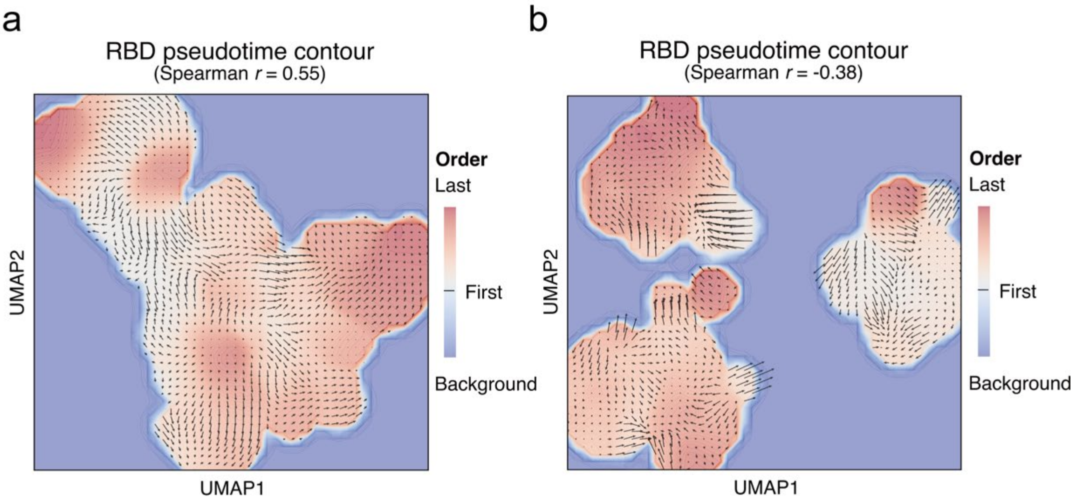

Supplementary Fig. 7 | Two-dimensional contour plot comparison. Ordering the sequences in pseudo time and visualize pseudo time values with contour plot. The contour color denotes the inferred sample time. Left, the contour plot with pseudo time inferred with our model. Right, the contour plot with pseudo time inferred with the ESM-1b model.

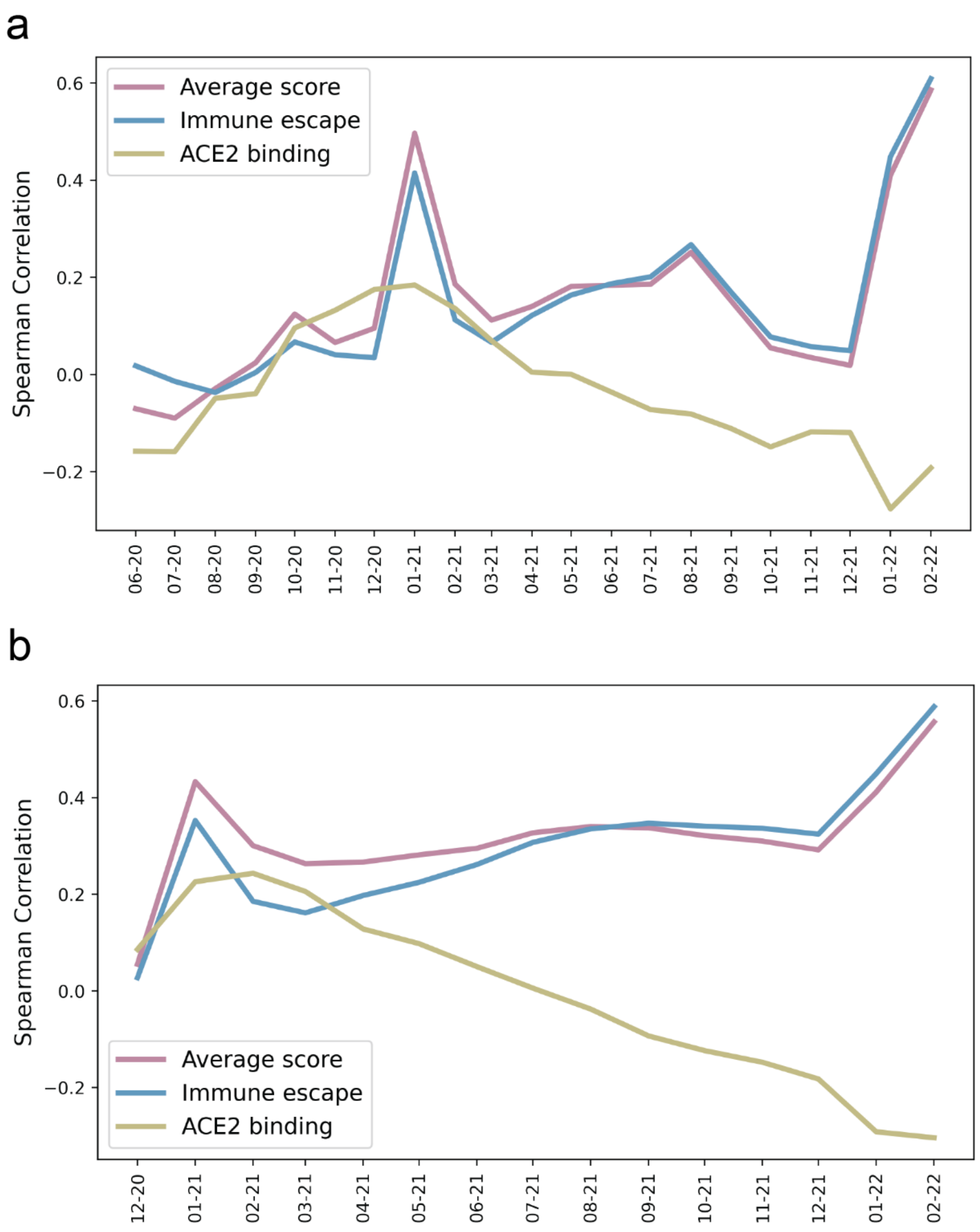

Supplementary Fig. 8 | Correlation between the model scores and sampling date. a, Spearman correlation overtime for the model predictions, including the ACE2 binding score, immune escape potential, and the weighted average of the two in a time window of previous six months for each sampled date. b, In a time window of previous 12 months for each sampled date.

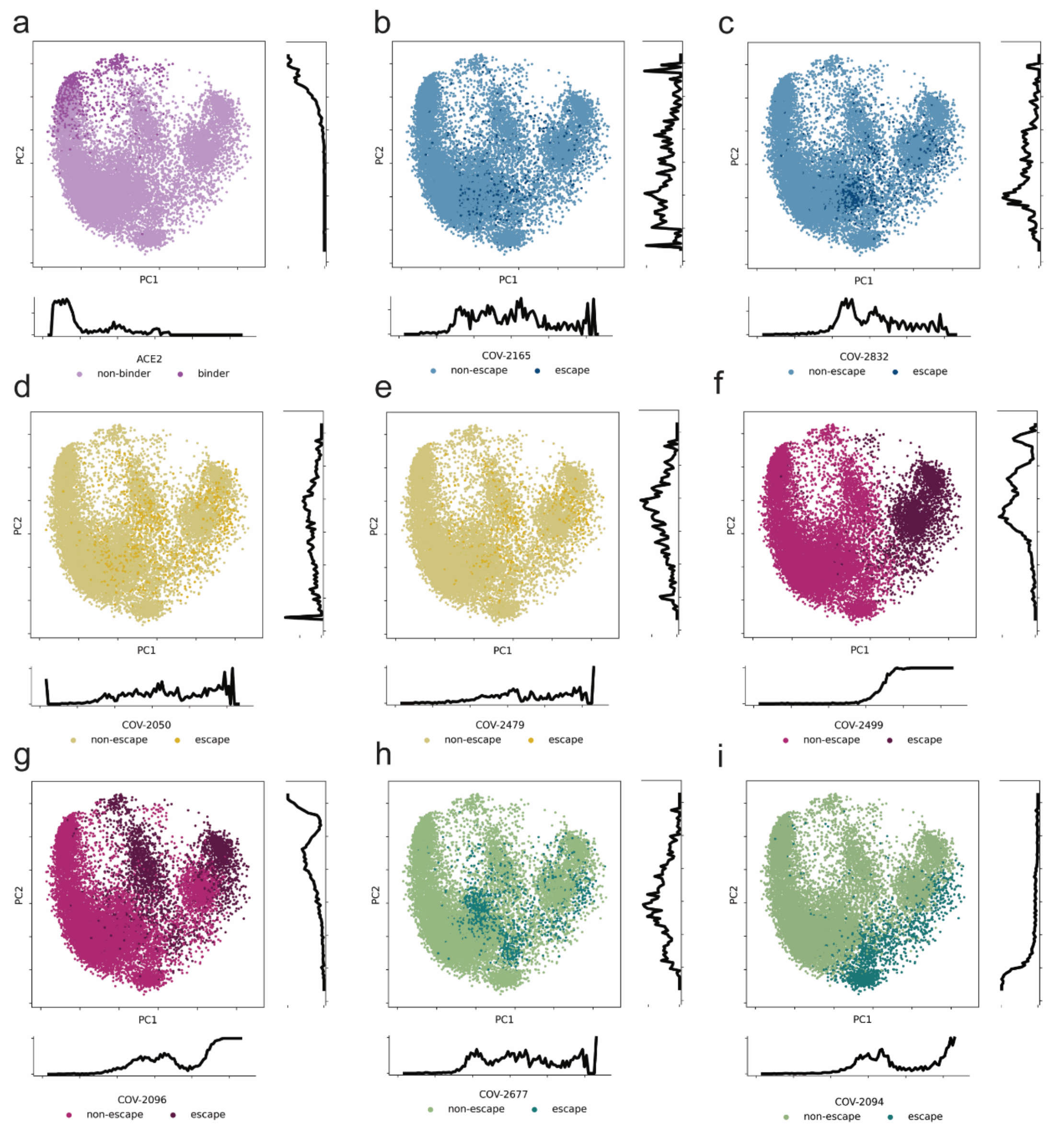

Supplementary Fig. 9 | Principal component analyses of the sequence's representations from our model. The detailed visualization of the model embeddings, colored by the escaping/binding ability towards ACE2 (purple), COV-2832, COV-2165(class 1 antibody, blue), COV-2479, COV-2500 (class 2 antibody, yellow), COV-2096, COV-2499 (class 3 antibody, red), COV-2677 and COV-2094 (class 4 antibody, green). Darker color denotes for binding(for ACE2) and escape (for antibodies), while light color denotes for non-binder (for ACE2) and non-escape(for antibodies).

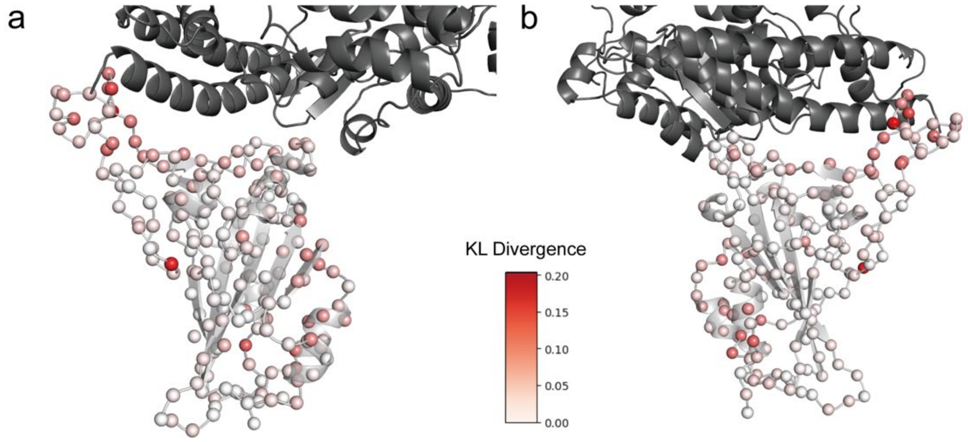

Supplementary Fig. 10 | 3D structure of RBD - ACE2 complex. The RBD structure is shown in as the ball-stick model while the ACE2 is colored in dark gray. The color of the RBD structure indicates the KL divergence between the initial seed sequences and the synthetic sequences, where red indicates higher KL divergence. a,b are viewed in different angles.

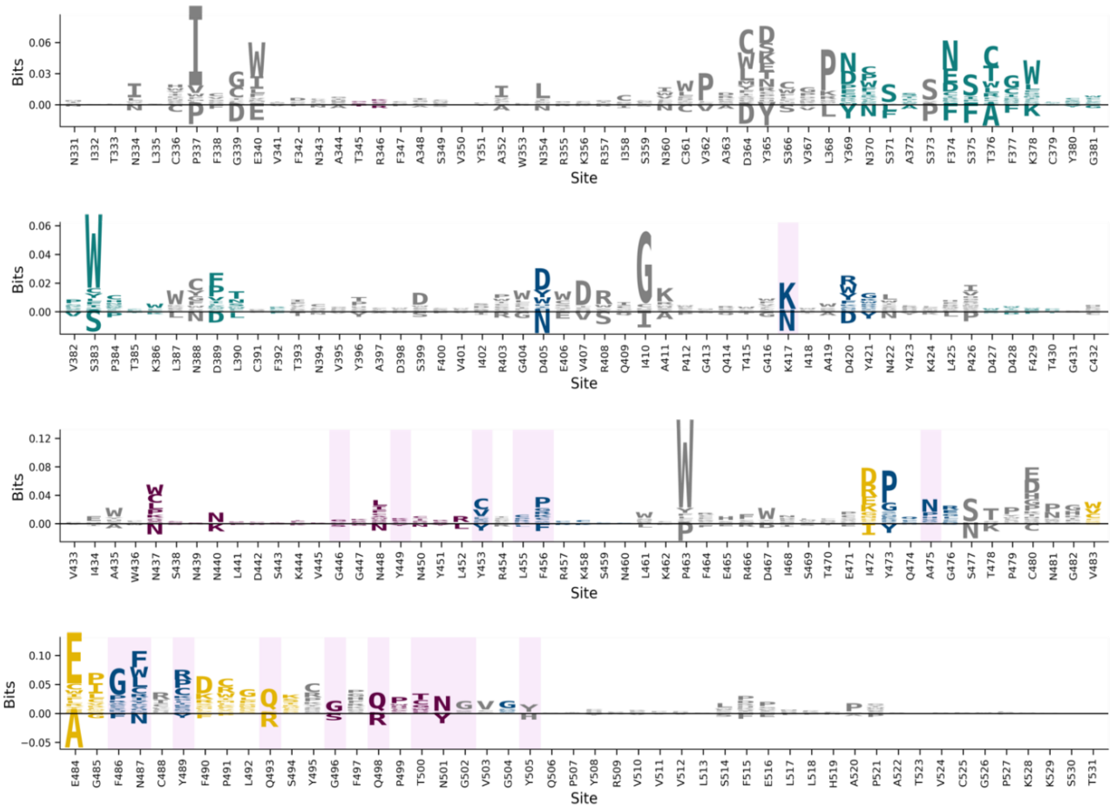

Supplementary Fig. 11 | KL logo plot for the entire RBD region. Sequence logos generated from the differences of the generated sequences and the initial sequences, spanning the entire RBD region. The logos were calculated with probability weighted Kullback-Leibler divergence with a pseudo count of 0.1.

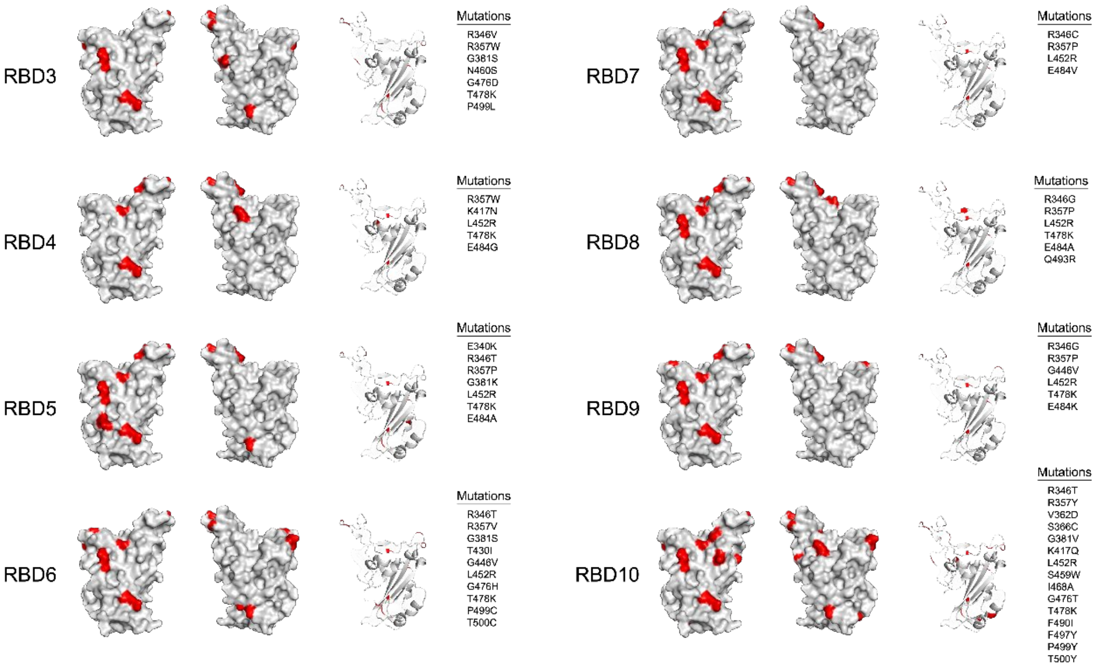

Supplementary Fig. 12 | Eight RBD mutants bearing different mutations on the surface were selected for binding assay against monoclonal antibodies. Surface modeling of mutant RBD proteins was illustrated in grey. Mutations sites were marked in red and listed beside the models.

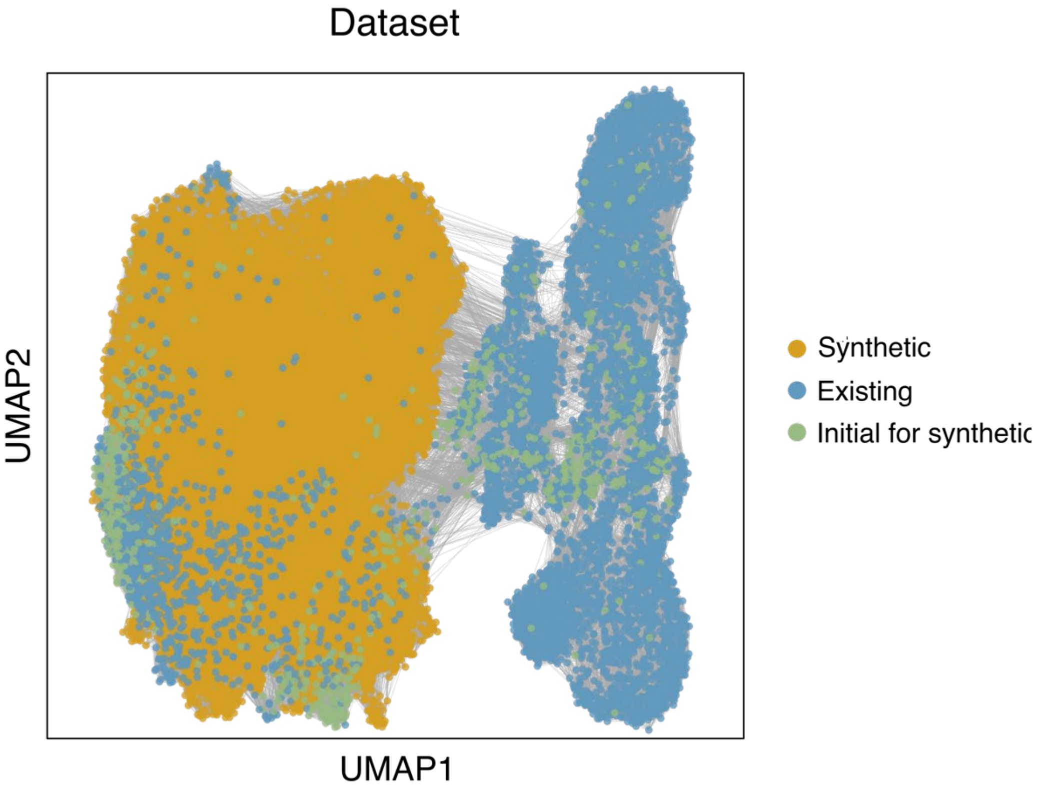

Supplementary Fig. 13 | Overview of the synthetic sequences and GISAID sequences. The landscape of synthetic RBD variant and GISAID variant sequences, represented as a KNN network and visualized use the UMAP, colored with the data origination. Blue color denotes for the existing RBD variant sequences from the GISAID database. Green denotes for a subset of

existing RBD variants that is used as seed sequences for generation. Yellow denotes for the synthetic RBD variant sequences.

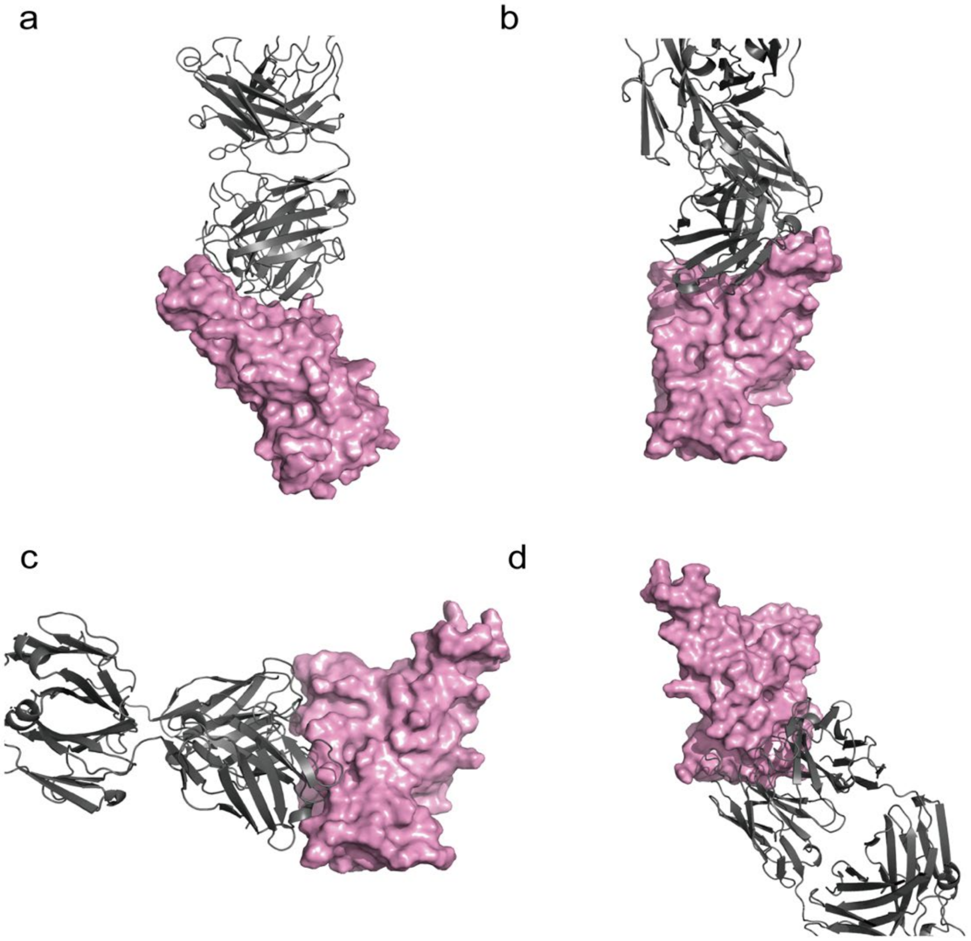

Supplementary Fig. 14 | 3D views of docking simulation results. The structure views for the *in silico* docking experiments. For each antibody and synthetic variant pair, there are 1000 docking results. RBD is colored in pink and antibodies are color by grey. a. One of the 1000 simulated docking results for LY-CoV16 and synthetic variant 1. b, One of the 1000 simulated docking results for LY-CoV555 and synthetic variant 1. c, One of the 1000 simulated docking results for S309 and synthetic variant 1. d, One of the 1000 simulated docking results for CR3022 and synthetic variant 1.

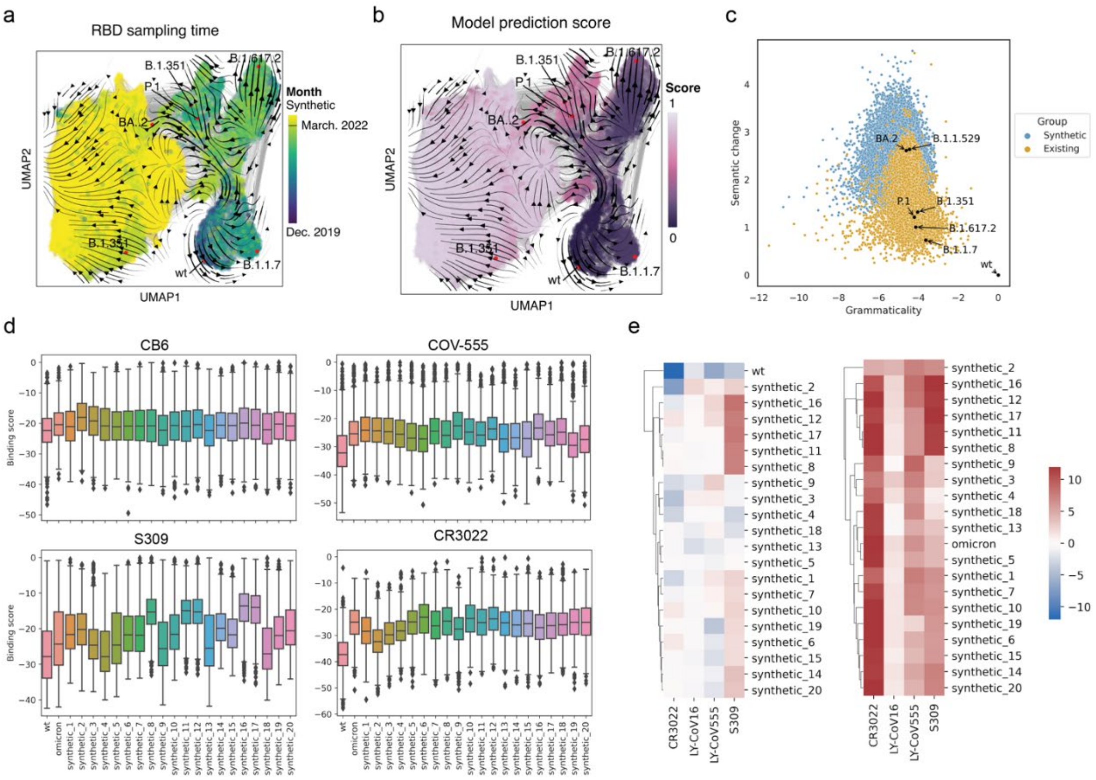

Supplementary Fig. 15| Evaluate the risks of the synthetic sequences. a, The landscape of existing SARS-COV-2 RBD variant (obtained from GISAID) and synthetic sequences, represented as a KNN-similarity graph. The gray lines indicate graph edges, the colored points denote the sequences with known sampling time/virtual time. The streamlines among the points show a visual trend from the existing sequences to synthetic sequences. b, The landscape of existing SARS-COV-2 RBD variant sequences (obtained from GISAID) and synthetic sequences, colored by model prediction score. c, The grammaticality and semantic analysis on existing GISAID sequences and synthetic sequences. d, The distribution of docking scores (n=1000) between four antibodies (CB6, S309, COV-555, CR3022) and RBD variants. Higher scores indicate better immune escape ability. The statistical significance is tested using student's *t*-test. The boxes represent the interquartile range, which spans from the 25th to the 75th percentiles, while the whiskers stretch to the maximum/minimum values that are within 1.5 times the interquartile range. Points outside this range are displayed as individual data points. e, Heatmap of the mean docking score difference. Red score suggests the variant sequence obtains higher immune escape ability compared with the Omicron (left) and Wuhan-wildtype(right).

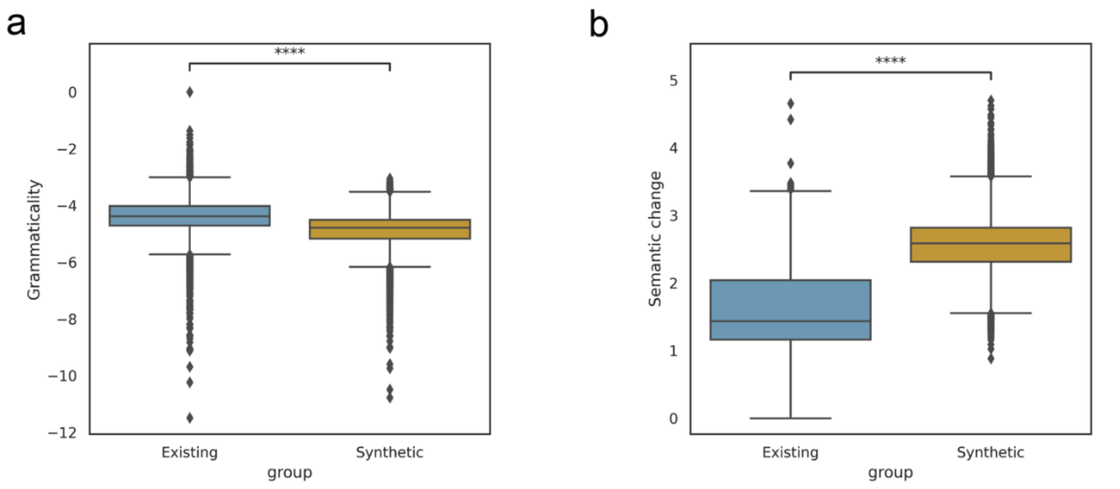

Supplementary Fig. 16 | Box plot for the existing GISAID sequences and the synthetic sequences. Grammatically and semantic change of the existing sequences (n=7594) and the synthetic sequences (n=38870) compared with that of the wild type Spike RBD sequence. Variant sequences with both high semantic change and high grammatically score is more likely to induce immune escape. In both plots, the boxes represent the interquartile range, which span from the 25th to the 75th percentiles, while the whiskers stretch to the maximum/minimum values that are within 1.5 times the interquartile range. Points outside this range are displayed as individual data points. '****' in the figure denotes that $p < 1.00e-04$ for two-sided student's t-test with Bonferroni correction. Due to the extremely small P values obtained in the case (approaching 0), we report these as $P < 1 \times 10^{-308}$ in the figure legends.

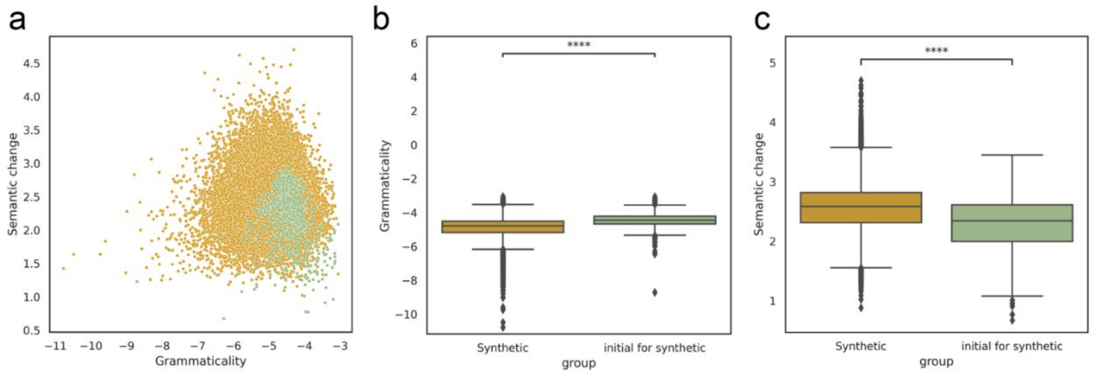

Supplementary Fig. 17 | The difference among the initial sequences and the synthetic sequences. a, Scatter plot represented the distribution of the of the existing sequences (n=7594) and the synthetic sequences (n=38870) in terms of the Grammatically and semantic change. b and c,

Box plot comparing the Grammatically and semantic change of the two groups. Both high semantic change and high grammatically score is more likely to induce immune escape. In both b and c plots, the boxes represent the interquartile range, which span from the 25th to the 75th percentiles, while the whiskers stretch to the maximum/minimum values that are within 1.5 times the interquartile range. Points outside this range are displayed as individual data points. '****' in the figure denotes that $p <= 1.00e-04$ for two-sided student's t-test with Bonferroni correlation. Due to the extremely small P values obtained in the case (approaching 0), we report these as $P < 1 \times 10^{-308}$ in the figure legends.

Supplementary Table. 1: Baseline model performance comparison

F1 score									
	ace2_bind	COV2-2096_400	COV2-2832_400	COV2-2094_400	COV2-2050_400	COV2-2677_400	COV2-2479_400	COV2-2165_400	COV2-2499_400
Our model	0.723	0.851	0.831	0.782	0.784	0.868	0.734	0.711	0.902
CNN	0.400	0.735	0.647	0.627	0.580	0.746	0.478	0.466	0.826
RNN	0.446	0.748	0.649	0.616	0.571	0.732	0.472	0.458	0.825
LSTM	0.366	0.721	0.669	0.587	0.588	0.743	0.478	0.470	0.823
Linear Regression	0.449	0.728	0.463	0.530	0.469	0.678	0.282	0.242	0.803
Random Forest	0.333	0.727	0.670	0.579	0.551	0.704	0.443	0.499	0.822
SVM	0.360	0.693	0.586	0.558	0.463	0.612	0.368	0.316	0.781
Linear Regression (Georiev)	0.440	0.702	0.456	0.505	0.464	0.675	0.291	0.264	0.770
Random Forest (Georiev)	0.396	0.718	0.662	0.577	0.553	0.697	0.424	0.496	0.814
SVM (Georiev)	0.360	0.693	0.587	0.559	0.463	0.612	0.368	0.314	0.781
Precision									
	ace2_bind	COV2-2096_400	COV2-2832_400	COV2-2094_400	COV2-2050_400	COV2-2677_400	COV2-2479_400	COV2-2165_400	COV2-2499_400
Our model	0.699	0.867	0.888	0.802	0.842	0.885	0.825	0.796	0.926
CNN	0.654	0.881	0.897	0.779	0.738	0.852	0.726	0.805	0.870
RNN	0.535	0.778	0.895	0.744	0.802	0.844	0.816	0.846	0.909
LSTM	0.552	0.905	0.824	0.850	0.832	0.838	0.766	0.759	0.894
Linear Regression	0.325	0.690	0.365	0.443	0.374	0.601	0.198	0.165	0.798
Random Forest	0.397	0.834	0.905	0.803	0.876	0.934	0.821	0.908	0.940
SVM	0.230	0.652	0.557	0.506	0.363	0.500	0.304	0.236	0.798
Linear Regression (Georiev)	0.326	0.649	0.362	0.416	0.382	0.628	0.214	0.192	0.744
Random Forest (Georiev)	0.432	0.777	0.844	0.732	0.755	0.849	0.645	0.821	0.908

SVM (Georiev)	0.230	0.652	0.560	0.507	0.363	0.500	0.304	0.235	0.797
Recall									
	ace2_bind	COV2-2096_400	COV2-2832_400	COV2-2094_400	COV2-2050_400	COV2-2677_400	COV2-2479_400	COV2-2165_400	COV2-2499_400
Our model	0.723	0.851	0.831	0.782	0.784	0.868	0.734	0.711	0.902
CNN	0.400	0.735	0.647	0.627	0.580	0.746	0.478	0.466	0.826
RNN	0.446	0.748	0.649	0.616	0.571	0.732	0.472	0.458	0.825
LSTM	0.366	0.721	0.669	0.587	0.588	0.743	0.478	0.470	0.823
Linear Regression	0.724	0.769	0.634	0.661	0.630	0.780	0.490	0.453	0.807
Random Forest	0.289	0.645	0.532	0.453	0.403	0.565	0.305	0.345	0.731
SVM	0.835	0.740	0.620	0.622	0.641	0.789	0.469	0.476	0.766
Linear Regression (Georiev)	0.680	0.765	0.616	0.643	0.593	0.731	0.458	0.425	0.799
Random Forest (Georiev)	0.366	0.667	0.546	0.477	0.437	0.591	0.317	0.357	0.737
SVM (Georiev)	0.835	0.740	0.620	0.622	0.641	0.789	0.469	0.475	0.766
Accuracy									
	ace2_bind	COV2-2096_400	COV2-2832_400	COV2-2094_400	COV2-2050_400	COV2-2677_400	COV2-2479_400	COV2-2165_400	COV2-2499_400
Our model	0.906	0.914	0.965	0.923	0.947	0.955	0.961	0.964	0.946
CNN	0.817	0.917	0.965	0.935	0.946	0.955	0.962	0.969	0.942
RNN	0.917	0.911	0.965	0.930	0.949	0.954	0.963	0.969	0.944
LSTM	0.920	0.916	0.964	0.934	0.951	0.955	0.963	0.968	0.943
Linear Regression	0.857	0.895	0.906	0.878	0.890	0.928	0.879	0.884	0.931
Random Forest	0.907	0.912	0.967	0.931	0.950	0.954	0.963	0.972	0.945
SVM	0.761	0.881	0.944	0.897	0.886	0.903	0.922	0.916	0.925
Linear Regression (Georiev)	0.861	0.882	0.906	0.868	0.895	0.932	0.892	0.903	0.917

Random Forest (Georiev)	0.909	0.905	0.965	0.927	0.946	0.950	0.958	0.971	0.941
SVM (Georiev)	0.761	0.881	0.944	0.897	0.886	0.903	0.922	0.916	0.925

Supplementary Table. 2. Validate model performance on extra datasets

Regression performance (Spearman Correlation coefficient) comparsion								
Dataset	Our model	CNN	RNN	LSTM	Georgiev linear regression	Linear regression	Random forest	SVM
A0A2Z5U3Z0_9INFA_Doud_2016	0.73521459	0.192456775	0.002335124	0.011438509	0.72231294	0.72271333	0.71822034	0.7256209
C6KNH7_9INFA_Lee_2018	0.75439883	0.192456775	-0.00742062	-0.00636827	0.76617646	0.76892648	0.76175894	0.76949613
IF1_ECOLI_Kelsic_2016	0.84472967	0.192456775	0.022604926	0.121341175	0.7138306	0.73234388	0.72994633	0.73875536
ADRB2_HUMAN_Jones_2020	0.62496298	0.064887272	-0.03620719	-0.07148929	0.43681035	0.45486954	0.44045849	0.45785954
MK01_HUMAN_Brenan_2016	0.54396553	0.192456775	-0.04189705	-0.03470799	0.38711399	0.38726715	0.42051833	0.41420424
BLAT_ECOLX_Ranganathan2015	0.85169547	0.192456775	-0.04189705	-0.03470799	0.75122153	0.76787681	0.74937014	0.76700267
ENV_HV1BR_Haddox_2016	0.28307102	0.064887272	-0.0066312	-0.00501729	-0.05383878	-0.0457555	0.01832819	-0.04502817
P53_HUMAN_Giacomelli_NULL_Etoposide_2018	0.71670701	0.192456775	-0.01850616	0.076586696	0.73697753	0.73877219	0.71158923	0.73562641
Classification performance (HER2 dataset) comparison								
Metrics	Our model	CNN	RNN	LSTM	Georgiev linear regression	Linear regression	Random forest	SVM
Accuracy	0.86	0.86	0.86	0.86	0.82	0.82	0.86	0.86
Precision	0.84	0.79	0.79	0.79	0.67	0.67	0.81	0.74
Recall	0.83	0.73	0.72	0.75	0.83	0.83	0.70	0.84

F1-score	0.84	0.76	0.76	0.77	0.74	0.74	0.75	0.79

Supplementary Table. 3 Model performance evaluation on antibody neutralization dataset

	antibody	antibody class	Spearman's correlation	pearson's correlation
0	CB6	Class 1	0.89822	0.885288
1	Brii-196	Class 1	0.738095	0.818321
2	20-Jan	Class 1	0.891631	0.856964
3	910-30	Class 1	0.864791	0.904059
4	REGN10933	Class 2	0.443122	0.858489
5	COV2-2196	Class 2	0.47619	0.827103
6	LY-CoV555	Class 2	0.866025	0.98812
7	15-Feb	Class 2	0.012276	0.432058
8	REGN10987	Class 3	0.634742	0.819847
9	COV2-2130	Class 3	0.670671	0.733331
10	S309	Class 3	0.590404	0.76496
11	7-Feb	Class 3	0.690476	0.759924
12	Brii-198	Class 3	0.214286	0.501515
13	ADG-2	Class 4	0.958101	0.746832
14	DH1047	Class 4	0.313276	0.726782
15	Oct-40	Class 4	0.934148	0.742667
16	S2X259	Class 4	0.807288	0.632627

Supplementary Table 4. Model predicted mutations was also found in immunocompromised patients

**Mutations**	**Case report time**	**Days post mAbs treatment**	**Region**	**Treatment**	**Reference**	**Note**
**E340K**	April 2022	11 days	France	sotrovimab	Sotrovimab drives SARS-CoV-2 omicron variant evolution in immunocompromised patients	
**S494P**	April 2022	35 days	US	bamlanivimab	SARS-CoV-2 evolution and immune escape in immunocompromised patients treated with exogenous antibodies	

**Q493R -> Q493**	April 2022	35 days	US	bamlanivimab	SARS-CoV-2 evolution and immune escape in immunocompromised patients treated with exogenous antibodies	
**F490S**	April 2021	56 days	Denmark	convalescent plasma	Persistent Severe Acute Respiratory Syndrome Coronavirus 2 Infection in Immunocompromised Host Displaying Treatment Induced Viral Evolution	Variant increases after received second time CP
**E484G**	Nov 2021	15 days	Germany	Ivermectin	Within-host evolution of SARS-CoV-2 in an immunosuppressed COVID-19 patient as a source of immune escape variants	Not treated by mab
**F490S**	Jan 2022	54 days	Spain	antiretroviral therapy	SARS-CoV-2 Evolution and Spike-Specific CD4+ T-Cell Response in Persistent COVID-19 with Severe HIV Immune Suppression	Not treated by mab
**P337L**	June 2022	24 days	Australia	sotrovimab	Resistance Mutations in SARS-CoV-2 Delta Variant after Sotrovimab Use	
**E340K**	June 2022	6/7 days (two cases)	Australia	sotrovimab	Resistance Mutations in SARS-CoV-2 Delta Variant after Sotrovimab Use	
**E340V**	June 2022	6/37 days (two cases)	Australia	sotrovimab	Resistance Mutations in SARS-CoV-2 Delta Variant after Sotrovimab Use	

**F490L, E484G, E484X,**					Recurrent SARS-CoV-2 Mutations in Immunodeficient Patients	COG-UK dataset
**Y489H**	June 2022	124 days	US	intermittent rituximab and eculizumab	Persistence and Evolution of SARS-CoV-2 in an Immunocompromised Host	
**G485R**	Feb 2022	~30 days	Israel	Antibody based treatment	Drivers of adaptive evolution during chronic SARS-CoV-2 infections	

Supplementary Table.5 In vitro validation experiments selection criteria

name	Lineages	RBD Mutations	seq	num_mut	why select it
Delta	B.1.617.2	L452R;T478K	NITNLCPFGEVFNATRFASVYAWNRKRISNCVADYSVLYNSASFSTFKCYGVSPTKLNDLCFTNVYADSFVIRGDEVRQIAPGQTGKIADYNYKLPDDFTGCVIAWNSNNLDSKVGGNYNYRYRLFRKSNLKPFERDISTEIYQAGSKPCNGVEGFNCYFPLQSYGFQPTNGVGYQPYRVVVLSFELLHAPATVCGPKKST	2	baseline
WT	WT		NITNLCPFGEVFNATRFASVYAWNRKRISNCVADYSVLYNSASFSTFKCYGVSPTKLNDLCFTNVYADSFVIRGDEVRQIAPGQTGKIADYNYKLPDDFTGCVIAWNSNNLDSKVGGNYNYLYRLFRKSNLKPFERDISTEIYQAGSTPCNGVEGFNCYFPLQSYGFQPTNGVGYQPYRVVVLSFELLHAPATVCGPKKST	0	baseline
RB	syn	R346V;R357W;G381S;N460S;	NITNLCPFGEVFNATVFASVYAWNRKWISNCVADYSVLYNSASFSTFKCYSVSPTKLNDLCFTNVYADSFVIRGDEVRQIAPGQTGKI	7	epistasis relationship. Linear model

D<br>3	t<br>h<br>e<br>ti<br>c	G476D;T478K;<br>P499L	ADYNYKLPDDFTGCVIAWNSNNLDSKVG<br>GNYNYLYRLFRKSSLKPFERDISTEIYQAD<br>SKPCNGVEGFNCYFPLQSYGFQLTNGVGY<br>QPYRVVVLSFELLHAPATVCGPKKST		disagree with ours on COV-2096, COV-2832, COV-2094, COV-2050, COV-2677, COV 2479,.COV2165, COV2499
R<br>B<br>D<br>4	s<br>y<br>n<br>t<br>h<br>e<br>ti<br>c	R357W;K417N;<br>L452R;T478K;<br>E484G	NITNLCPFGEVFNATRFASVYAWNRKWIS<br>NCVADYSVLYNSASFSTFKCYGVSPTKLN<br>DLCFTNVYADSFVIRGDEVRQIAPGQTGNI<br>ADYNYKLPDDFTGCVIAWNSNNLDSKVG<br>GNYNYRYRLFRKSNLKPFERDISTEIYQA<br>GSKPCNGVGGFNCYFPLQSYGFQPTNGV<br>GYQPYRVVVLSFELLHAPATVCGPKKST	5	Epistasis relationship. Linear model disagree with ours on COV-2832, COV-2094, COV-2677, COV 2479,.COV2165, COV2499
R<br>B<br>D<br>5	s<br>y<br>n<br>t<br>h<br>e<br>ti<br>c	E340K;R346T;<br>R357P;G381K;<br>L452R;T478K;<br>E484A	NITNLCPFGKVFNATTFASVYAWNRKPISN<br>CVADYSVLYNSASFSTFKCYKVSPTKLND<br>LCFTNVYADSFVIRGDEVRQIAPGQTGKIA<br>DYNYKLPDDFTGCVIAWNSNNLDSKVGG<br>NYNYRYRLFRKSNLKPFERDISTEIYQAGS<br>KPCNGVAGFNCYFPLQSYGFQPTNGVGY<br>QPYRVVVLSFELLHAPATVCGPKKST	7	Epistasis relationship. Linear model disagree with ours on COV-2832, COV-2094, COV-2677, COV 2479,.COV2165, COV2499
R<br>B<br>D<br>6	s<br>y<br>n<br>t<br>h<br>e<br>ti<br>c	'R346T;R357V;<br>G381S;T430I;G<br>446V;L452R;G<br>476H;T478K;P<br>499C;T500C'	NITNLCPFGEVFNATTFASVYAWNRKVISN<br>CVADYSVLYNSASFSTFKCYSVSPTKLND<br>LCFTNVYADSFVIRGDEVRQIAPGQTGKIA<br>DYNYKLPDDFIGCVIAWNSNNLDSKVVG<br>NYNYRYRLFRKSNLKPFERDISTEIYQAHS<br>KPCNGVEGFNCYFPLQSYGFQCCNGVGY<br>QPYRVVVLSFELLHAPATVCGPKKST	1<br>0	Long mutations,epistasis relationship. Linear model disagree with ours on COV 2832, Cov-2050, COV-2094, COV-2677, COV

					2479,.COV21 65
RBD7	synthetic	R346C;R357P; L452R;E484V	NITNLCPFGEVFNATCFASVYAWNRKPISNCVADYSVLYNSASFSTFKCYGVSPTKLNDLCFTNVYADSFVIRGDEVRQIAPGQTGKIADYNYKLPDDFTGCVIAWNSNNLDSKVGGNYNYRYRLFRKSNLKPFERDISTEIYQAGSTPCNGVVGFNCYFPLQSYGFQPTNGVGYQPYRVVVLSFELLHAPATVCGPKKST	4	Small number of mutations. Linear model disagree with ours on COV-2832, COV-2094, COV-2677, COV 2479,.COV21 65, COV2499
RBD8	synthetic	R346G;R357P; L452R;T478K; E484A;Q493R	NITNLCPFGEVFNATGFASVYAWNRKPISNCVADYSVLYNSASFSTFKCYGVSPTKLNDLCFTNVYADSFVIRGDEVRQIAPGQTGKIADYNYKLPDDFTGCVIAWNSNNLDSKVGGNYNYRYRLFRKSNLKPFERDISTEIYQAGSKPCNGVAGFNCYFPLRSYGFQPTNGVGYQPYRVVVLSFELLHAPATVCGPKKST	6	Mutations similar to omicron.
RBD9	synthetic	R346G;R357P; G446V;L452R; T478K;E484K	NITNLCPFGEVFNATGFASVYAWNRKPISNCVADYSVLYNSASFSTFKCYGVSPTKLNDLCFTNVYADSFVIRGDEVRQIAPGQTGKIADYNYKLPDDFTGCVIAWNSNNLDSKVVGNYNYRYRLFRKSNLKPFERDISTEIYQAGSKPCNGVKGFNCYFPLQSYGFQPTNGVGYQPYRVVVLSFELLHAPATVCGPKKST	6	Largest linear model predicted escape value
RBD10	synthetic	R346T;R357Y; V362D;S366C; G381V;K417Q; L452R;S459W; I468A;G476T;T478K;F490I;F497Y;P499Y;T500Y	NITNLCPFGEVFNATTFASVYAWNRKYISNCDADYCVLYNSASFSTFKCYVVSPTKLNDLCFTNVYADSFVIRGDEVRQIAPGQTGQIADYNYKLPDDFTGCVIAWNSNNLDSKVGGNYNYRYRLFRKWNLKPFERDASTEIYQATSKPCNGVEGFNCYIPLQSYGYQYYNGVGYQPYRVVVLSFELLHAPATVCGPKKST	15	Largest deep learning model predicted escape value

References:

1. Swanson, K., Chang, H. & Zou, J. Predicting Immune Escape with Pretrained Protein Language Model Embeddings. 2022.2011.2030.518466 (2022).
