# Supplementary material for: Predicting the antigenic evolution of SARS-COV-2 with deep learning
Source: Nat Commun. 2023 Jun 13;14:3478. doi: 10.1038/s41467-023-39199-6 (PMC10261845; doi:10.1038/s41467-023-39199-6)
Supplement: Supplementary file 3 — Reporting Summary [file 41467_2023_39199_MOESM3_ESM.pdf]

nature portfolio

Corresponding author(s): Xin Gao

Last updated by author(s): Jul 1, 2022

Reporting Summary

Nature Portfolio wishes to improve the reproducibility of the work that we publish. This form provides structure for consistency and transparency in reporting. For further information on Nature Portfolio policies, see our Editorial Policies and the Editorial Policy Checklist.

Statistics

For all statistical analyses, confirm that the following items are present in the figure legend, table legend, main text, or Methods section.

n/a	Confirmed	
☐	☒	The exact sample size (*n*) for each experimental group/condition, given as a discrete number and unit of measurement
☐	☒	A statement on whether measurements were taken from distinct samples or whether the same sample was measured repeatedly
☐	☒	The statistical test(s) used AND whether they are one- or two-sided<br>*Only common tests should be described solely by name; describe more complex techniques in the Methods section.*
☒	☐	A description of all covariates tested
☒	☐	A description of any assumptions or corrections, such as tests of normality and adjustment for multiple comparisons
☐	☒	A full description of the statistical parameters including central tendency (e.g. means) or other basic estimates (e.g. regression coefficient) AND variation (e.g. standard deviation) or associated estimates of uncertainty (e.g. confidence intervals)
☐	☒	For null hypothesis testing, the test statistic (e.g. *F*, *t*, *r*) with confidence intervals, effect sizes, degrees of freedom and *P* value noted<br>*Give P values as exact values whenever suitable.*
☒	☐	For Bayesian analysis, information on the choice of priors and Markov chain Monte Carlo settings
☒	☐	For hierarchical and complex designs, identification of the appropriate level for tests and full reporting of outcomes
☒	☐	Estimates of effect sizes (e.g. Cohen's *d*, Pearson's *r*), indicating how they were calculated

Our web collection on statistics for biologists contains articles on many of the points above.

Software and code

Policy information about availability of computer code

Data collection	The deep mutational scanning datasets is publicly available at https://jbloomlab.github.io/SARS-CoV-2-RBD_DMS/ https://github.com/jbloomlab/SARS-CoV-2-RBD_DMS/blob/master/results/binding_Kds/binding_Kds.csv and https://media.githubusercontent.com/media/jbloomlab/SARS-CoV-2-RBD_MAP_Crowe_antibodies/master/results/escape_scores/scores.csv . The pseudovirus neutralization test assay data is publicly available in its original publications. The GISAID hCoV-19 variants sequence data is publicly available at https://www.gisaid.org/. We retrieved data from the website on 8 March 2022. The PDB data was used for visualization and docking experiments, we used: PDB id: 6m0j [http://doi.org/10.2210/pdb6m0j/pdb]; PDB id: 7c01 [http://doi.org/10.2210/pdb7c01/pdb]; PDB id: 7kMG [http://doi.org/10.2210/pdb7kMG/pdb]; PDB id: 7R6W [http://doi.org/10.2210/pdb7R6W/pdb]; PDB id: 6w41 [http://doi.org/10.2210/pdb6w41/pdb]. The generated variant sequences and other source data are provided as a Source Data file.
Data analysis	We compared our multi-task model with gUnirep(https://github.com/churchlab/UniRep), eUnirep(https://github.com/churchlab/UniRep), and augmented potts model(https://github.com/chloechsu/combining-evolutionary-and-assay-labelled-data), following their github repository. We performed Evo-velocity analysis with https://evolocity.readthedocs.io/en/latest/. Besides, we visualized our model embeddings with UMAP version 0.5. We performed docking experiments with SnugDock in Rosetta 3. We visualized the protein structures with PyMol 2.4. We use Sklearn version 1.1.1 and Scipy65 1.6.0 for measuring model performance. The source code for this study can be accessed at the GitHub repository: https://github.com/WHan-alter/MLAEP. The webserver can be found at https://mlaep.cbrc.kaust.edu.sa/. A permanent archive of the source code is also available on Zenodo at https://doi.org/10.5281/zenodo.778186766.

For manuscripts utilizing custom algorithms or software that are central to the research but not yet described in published literature, software must be made available to editors and reviewers. We strongly encourage code deposition in a community repository (e.g. GitHub). See the Nature Portfolio guidelines for submitting code & software for further information.

Data

Policy information about availability of data

All manuscripts must include a data availability statement. This statement should provide the following information, where applicable:

- Accession codes, unique identifiers, or web links for publicly available datasets
- A description of any restrictions on data availability
- For clinical datasets or third party data, please ensure that the statement adheres to our policy

The deep mutational scanning datasets is publicly available at https://jbloomlab.github.io/SARS-CoV-2-RBD_DMS/ https://github.com/jbloomlab/SARS-CoV-2-RBD_DMS/blob/master/results/binding_Kds/binding_Kds.csv and https://media.githubusercontent.com/media/jbloomlab/SARS-CoV-2-RBD_MAP_Crowe_antibodies/master/results/escape_scores/scores.csv . The pseudovirus neutralization test assay data is publicly available in its original publications. The GISAID hCoV-19 variants sequence data is publicly available at https://www.gisaid.org/. We retrieved data from the website on 8 March 2022. The PDB data was used for visualization and docking experiments, we used: PDB id: 6m0j [http://doi.org/10.2210/pdb6m0j/pdb]; PDB id: 7c01 [http://doi.org/10.2210/pdb7c01/pdb]; PDB id: 7kMG [http://doi.org/10.2210/pdb7kMG/pdb]; PDB id: 7R6W [http://doi.org/10.2210/pdb7R6W/pdb]; PDB id: 6w41 [http://doi.org/10.2210/pdb6w41/pdb]. The generated variant sequences and other source data are provided as a Source Data file.

Human research participants

Policy information about studies involving human research participants and Sex and Gender in Research.

Reporting on sex and gender	N/A. Since HRP is not applicable to the study
Population characteristics	N/A. Since HRP is not applicable to the study
Recruitment	N/A. Since HRP is not applicable to the study
Ethics oversight	N/A. Since HRP is not applicable to the study

Note that full information on the approval of the study protocol must also be provided in the manuscript.

Field-specific reporting

Please select the one below that is the best fit for your research. If you are not sure, read the appropriate sections before making your selection.

[x] Life sciences [ ] Behavioural & social sciences [ ] Ecological, evolutionary & environmental sciences

For a reference copy of the document with all sections, see nature.com/documents/nr-reporting-summary-flat.pdf

Life sciences study design

All studies must disclose on these points even when the disclosure is negative.

Sample size	No formal sample size calculation was performed for this study. The sample sizes for each dataset were determined based on the availability of data and the objective of achieving a representative and diverse set of sequences for training, validation, and testing. We collected 19,132 samples from Deep Mutational Scanning experiments to train and validate our multi-task model, which provided a large and diverse dataset to capture various sequence-phenotype relationships. For the independent test sets, we used 15 sequences from the pVNT dataset and 7,594 sequences from the GISAID database, which allowed us to evaluate the model's performance on datasets with different characteristics. The consistent performance of the model across these datasets suggests that the chosen sample sizes are sufficient for the purposes of this study.
Data exclusions	The pVNT dataset and GISAID dataset contains sequences spanning the entire Spike protein, we kept the RBD region, and removed the duplicates as we only focused on the unique RBD region in this study.<br>The HTRF-based antigen-antibody binding assay excluded data points with significant hook effect from the dose-response curve fitting.
Replication	For model training, we conducted five-fold cross-validation and reported the average performance and the corresponding variance. In addition, we reproduced the docking experiments for 1000 times.<br>The HTRF-based antigen-antibody binding assay were performed independently for three times, and all attempts at replication were successful.
Randomization	We first shuffled all samples in our datasets after data preprocessing procedure. After creating the benchmark dataset for training the multi-task model, we used five-fold cross-validation to perform random splitting. When generating the synthetic sequences, we added random seeds to ensure the next generations have variances. When performing docking experiments, antibodies and antigens were randomly placed to avoid bias.<br>Randomization was not relevant in the HTRF-based antigen-antibody binding assay, because samples were not allocated in groups.
Blinding	The investigators were blinded to group allocation.<br>Blinding was not performed in HTRF-based antigen-antibody binding assay, because it is not a common procedure for the method employed

Reporting for specific materials, systems and methods

We require information from authors about some types of materials, experimental systems and methods used in many studies. Here, indicate whether each material, system or method listed is relevant to your study. If you are not sure if a list item applies to your research, read the appropriate section before selecting a response.

Materials & experimental systems

n/a	Involved in the study
☐	☒ Antibodies
☐	☒ Eukaryotic cell lines
☒	☐ Palaeontology and archaeology
☒	☐ Animals and other organisms
☒	☐ Clinical data
☒	☐ Dual use research of concern

Methods

n/a	Involved in the study
☒	☐ ChIP-seq
☒	☐ Flow cytometry
☒	☐ MRI-based neuroimaging

Antibodies

Antibodies used	The previously described human anti-SARS-COV-2 RBD monoclonal neutralizing antibodies (COV2-2050, COV2-2499, COV2-2096, COV2-2479, COV2-2832, COV2-2165, COV2-2094, COV2-2677, from PMID: 32651581) were ordered from GeneScript Biotech (TurboCHO-HT Recombinant Antibody Service). These recombinant antibodies were not commercially available.<br>Monoclonal antibody anti-6His-Tb-cryptate Gold (Cisbio 61HI2TLA, Lot 19A) was used as the HTRF donor for the RBD variant-neutralizing antibody binding assay.<br>Goat anti-Human IgG (H+L) Cross-Adsorbed Secondary Antibody, Alexa Fluor 647 (Thermo Fisher A-21445, Lot 2491370) was used as the HTRF acceptor for the RBD variant-neutralizing antibody binding assay.
Validation	All commercially available antibodies are validated by manufacturers. Additionally information can be found on product website and listed below.<br>https://assets.thermofisher.com/TFS-Assets/BID/certificate/Certificates-of-Analysis/A21445%20Lot%202491370%20CofA.pdf<br>https://www.cisbio.net/media/asset/c/i/cisbio_dd_bi_20250112_61hi2tla_19a.pdf<br>Validation of human anti-SARS-COV-2 RBD monoclonal neutralizing antibodies were previously described (PMID: 32651581).

Eukaryotic cell lines

Policy information about cell lines and Sex and Gender in Research

Cell line source(s)	Expi293F (ThermoFisher Scientific, A14527)
Authentication	The cell line used was not authenticated
Mycoplasma contamination	Cell lines used were not tested for mycoplasma contamination
Commonly misidentified lines<br>(See ICLAC register)	No commonly misidentified cell lines were used
